# Purification of Recombinant Peanut Allergen Ara h 1 and Comparison of IgE Binding to the Natural Protein

**DOI:** 10.3390/foods3040642

**Published:** 2014-12-18

**Authors:** Barry K. Hurlburt, Jane K. McBride, Jacqueline B. Nesbit, Sanbao Ruan, Soheila J. Maleki

**Affiliations:** Southern Regional Research Center, Agricultural Research Service, United States Department of Agriculture, 1100 Robert E. Lee, Blvd., New Orleans, LA 70124, USA; E-Mails: jane.mcbride@ars.usda.gov (J.K.M.); jbnesbit@uno.edu (J.B.N.); cyoumc@yahoo.com (S.R.); soheila.maleki@ars.usda.gov (S.J.M.)

**Keywords:** peanut allergy, immunodominant, Ara h 1, IgE binding

## Abstract

Allergic reactions to food are on the rise worldwide and there is a corresponding increase in interest to understand the molecular mechanisms responsible. Peanut allergies are the most problematic because the reaction often persists into adulthood and can be as severe as anaphylaxis and death*.* The purpose of the work presented here was to develop a reproducible method to produce large quantities of pure recombinant Ara h 1(rAra h 1) that will enable standardization of immunological tests for patients and allow structural and immunological studies on the wild type and mutagenized forms of the protein. Ara h 1 is initially a pre-pro-protein which, following two endoproteolytic cleavages, becomes the mature form found in peanut. The mature form however has flexible regions that make it refractory to some structural studies including crystallography. Therefore, independent purification of the mature and core regions was desirable. Expression constructs were synthesized cDNA clones for each in a pET plasmid vector without tags. Codons were optimized for expression in *E. coli*. High-level expression was achieved in BL21 strains. Purification to near homogeneity was achieved by a combination of ammonium sulfate precipitation and ion exchange chromatography. The purified rAra h 1 was then compared with natural Ara h 1 for IgE binding. All patients recognized both the folded natural and rAra h 1, but the IgE binding to the rArah1 was significantly reduced in comparison to the natural allergen, which could potentially make it useful for immunotherapeutic purposes.

## 1. Introduction

Peanuts (*Arachis hypogaea*) are a tremendously important food with over 30 million metric tons harvested a year worldwide (US Department of Agriculture, [1]). They contain 25% protein comprised of 18 of the 20 naturally occurring amino acids (not asparagine or glutamine) including all of the essential amino acids and are nearly 50% oil. Their high nutritional value and low production cost has made peanut one of the most prevalent foods in the world. They are eaten intact, as a butter, and as additives in a wide variety of processed foods from candy to canned chili. Unfortunately, peanuts are also a frequent cause of food allergy and can cause severe reactions, including fatal anaphylaxis. In the United States more than 1% of the population is peanut allergic [2]. There are currently no good therapies for peanut allergy; avoidance is the only option for patients.

The protein composition of a peanut is almost entirely a small number of seed storage proteins and these are the predominant allergens. Three of these Ara h 1, Ara h 2, and Ara h 3 are immunodominant, in that the majority of peanut allergic patient’s sera contain IgE antibodies specific for these proteins [3,4,5]. Considerable research over many years has resulted in a wealth of knowledge about these antigens. Ara h 1 is a vicilin and member of the 7S globulin family [4] and makes up approximately 15% of the peanut [6]. It is a trimer comprised of ~67 kDa subunits [4,6]. The linear IgE epitopes have been mapped by several groups [4,7,8,9]. Roasting is the most common processing treatment for peanuts prior to consumption and this procedure heightens the allergenicity of Ara h 1 [6]. During roasting sugars modify the proteins or are attached to the protein via the Maillard reaction. Furthermore, roasting results in an Ara h 1 protein that is less digestible by gastrointestinal enzymes [6].

For several projects that our group has planned having a reproducible system to express recombinant Ara h 1 to high levels and purify it is required. One of those projects is to have a constant source of identical protein to use in development of diagnostic systems to determine peanut allergy. We are also interested in pursuing structural studies with the wild type and mutant forms of Ara h 1. As mentioned above, the linear epitopes bound by IgE were mapped. In addition, individual amino acids within those epitopes were changed and significant reduction in IgE binding could be achieved by one or two substitutions [4]. It is possible that a recombinant mutant Ara h 1 with reduced IgE binding could be developed and may be useful in immunotherapy.

**Figure 1:**
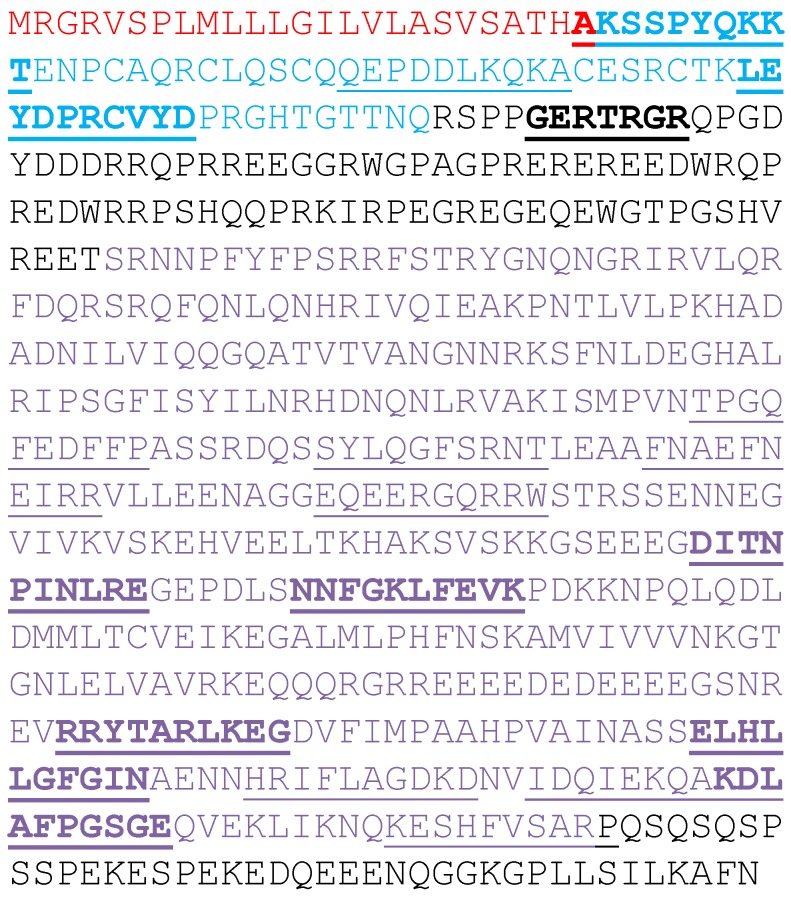
Amino acid sequence of Ara h 1. The entire primary translation product is shown. In red is the sequence of a leader sequence that is proteolytically removed. In blue is the signal sequence that is also proteolytically removed. In black and purple is the mature Ara h 1 found in peanut. In purple is the Ara h 1 core sequence. The linear epitopes identified by others are underlined. The immunodominant epitopes are also shown in bold typeface.

Ara h 1 is translated as a pre-pro-protein (Figure 1). A signal peptide (red in Figure 1) presumably directs the nascent protein to the vacuole prior to its cleavage. There is also a leader sequence (blue in Figure 1) of unknown function that is removed yielding the mature Ara h 1 protein (black and purple in Figure 1) [10]. Interestingly, three immunodominant epitopes were mapped to this leader sequence [11]. A pET derived construct of the mature Ara h 1 coding region was made. Since it has been reported that the *N*- and *C*-terminal extensions (black in Figure 1) of a highly-conserved core domain are flexible and inhibit crystal formation [12,13,14], we also generated a pET expression construct of the core domain alone (purple in Figure 1).

## 2. Experimental Section

### 2.1. Plasmid Constructions and Protein Expression

The initial translation product of Ara h 1 is shown in Figure 1. The mature protein found in peanut starts at the arginine at position 85 and ends with the asparagine at position 626 (shown in black and purple in Figure 1). The mature protein sequence was back translated into codons preferred for expression in *E. coli* using programs resident in DNASTAR’s Lasergene software (Madison, WI, USA). EZBiolab (Carmel, IN, USA) synthesized the gene and added CATATG to the 5′ end for an Nde I restriction site and start methionine and TAAGGATCC to the 3′ end for a Bam HI restriction site and stop codon. The synthetic gene was excised with Nde I and Bam HI and cloned into equally cut pET9a yielding pET-rAra h 1. A shorter core region (codons 170–586) was subsequently amplified using the synthetic gene as a template in PCR using primers: 5′-GGAGCCCATATGTCTCGTAACAACCCGTTCTACTT-3′ and 5′-GGAGCCGGATCCTTAACGAGCAGAAACGAAGTGA-3′. The PCR product was then digested with Nde I and Bam HI and cloned into pET9a yielding pET-rsAra h 1. The fidelity of pET-rsAra h 1 was confirmed by DNA sequencing. To express these recombinant proteins, the plasmids were transformed into BL21 (DE3) cells. Individual colonies were inoculated and grown overnight at 37 °C in 5 mL of Luria-Bertani (LB) broth containing 25 μg/mL kanamycin. Five hundred milliliters cultures in 2 L baffle flasks were inoculated with 500 μL of the overnight culture. Growth was monitored by absorbance at 600 nm. IPTG (isopropyl-β-d-thiogalactopyranoside) was added to 1 mM when the culture reached 0.6 O.D. to induce protein expression. Cells that were induced for 3 h were harvested by centrifugation at 5200× *g* at 4 °C for 10 min.

### 2.2. Purification of Mature rAra h 1

Wet cell paste from L of culture was stored at −20 °C until needed. Cells were thawed on ice then resuspended in 100 mL of SJM-400 buffer (50 mM Tris-Cl, pH 8.4, 1 mM EDTA, 1 mM PMSF, 400 mM NaCl). Two hundred microliters of lysozyme solution (50 mg/mL) was added and held on ice until the solution was viscous indicating cell lysis. The solution was treated with ultrasound on ice until all viscosity was removed. Insoluble material was removed by centrifugation at 17,500× *g*, 4 °C, 30 min. Solid ammonium sulfate was added slowly to the supernatant with stirring at 4 °C to 25% of saturation and stirred further for 30 min. Precipitated material was removed by centrifugation at 17,500× *g*, 4 °C, 30 min. Ammonium sulfate was added to the supernatant to 50% of saturation, stirred and centrifuged as above. The pellet was dissolved in 50 mL of Q column buffer (50 mM Tris-Cl, pH 8.4, 1 mM EDTA) and loaded onto a pre-equilibrated High-Q column (2.5 × 10 cm) (Bio-Rad, Hercules, CA, USA). The column was washed with the same buffer until the optical density (280 nm) of the effluent was zero. The column was further washed with the same buffer containing 100 mM NaCl. The bound rAra h 1 was eluted with a linear gradient of 100–250 mM NaCl in Q column buffer.

### 2.3. Purification of Core rAra h 1

The cells were lysed exactly as for the mature rAra h 1, except that 0.4% Triton X-100 was present in the lysis buffer. To the cleared lysate solid ammonium sulfate was added to 75% of saturation and cleared as described above. Ammonium sulfate was then added to the supernatant to 100%, stirred 30 min and the precipitate collected by centrifugation. The precipitate was dissolved in 50 mL of Q column buffer and loaded onto a High-Q column. The column was washed with the same buffer until the optical density (280 nm) of the effluent was zero. rsAra h 1was eluted with Q column buffer containing 100 mM NaCl. The eluate was adjusted to pH 7.0 with HCl and NaCl to a final concentration of 280 mM. This was loaded onto a High-S column (Bio-Rad) that was equilibrated with S column buffer (50 mM Tris-Cl, pH 7.0, 1 mM EDTA) with 280 mM NaCl. The column was washed with that buffer until the absorbance at 280 nm was zero. rsAra h 1 was eluted with 450 mM NaCl in column buffer. The High-S column was washed with buffer containing 2 M NaCl, then re-equilibrated with S column buffer with 300 mM NaCl. The eluate was diluted with S column buffer with no salt to give a final NaCl concentration of 300 mM and re-loaded on the column. Short rAra h 1 was eluted with a linear gradient of 300 mM–2 M NaCl in column buffer.

### 2.4. Gel Electrophoresis and Western Blots

For gel analysis protein samples were mixed with 6× sample buffer and heated to 100 °C for 10 min. Proteins were resolved on commercial 4%–20% gradient gels (Bio-Rad). The molecular weight markers for stained gels were SeeBlue+2 (Invitrogen, Carlsbad, CA, USA) and for western blots Magic Mark 2 (Invitrogen). To visualize the proteins the gels were stained with Gel-Code Blue (Pierce, Rockford, IL, USA). For western blotting, proteins were electrophoretically transferred to PVDF membranes. Membranes were blocked for 1 h at room temperature with 5% non-fat dry milk in PBST (phosphate-buffered saline, 0.05% Tween-20) followed by chicken anti-Ara h 1 (1:5000). After washing, antibody binding was detected with horseradish peroxidase-conjugated anti-chicken IgG (1:100,000) in 2% non-fat dry milk in PBST and ECL Plus chemiluminesence following the manufacturer’s instructions (GE Healthcare, Piscataway, NJ, USA). A FujiFilm LAS-1000 recorded the signal.

### 2.5. Secondary Structure Determination 

Far UV (185–250 nm) circular dichroism (CD) spectra of purified rAra h 1, rsAra h 1 and natural Ara h 1 (purified from peanut; [15]) were obtained. Samples were desalted using disposable gel filtration columns (G-25, PD-10, GE Healthcare, Uppsala, Sweden) into Milli-Q water and immediately used in CD measurements. A CD spectrum of Milli-Q water was obtained for background purposes and subtracted from each spectra. Protein concentrations were 0.1 mg/mL and spectra were obtained at room temperature with a JASCO Model J-710 spectropolarimeter (JASCO, Easton, MD, USA). Secondary structural modes were estimated from ellipticities by multiple protein secondary structure prediction and calculation programs such as K2d using a Kohonen neural network with a 2-dimensional output layer [16], Sspro [16], PSIPRED (http://bioinf.cs.ucl.ac.uk/cgi-bin/psipred), PROFsec [17,18], CDPro (http://lamar.colostate.edu/~sreeram/CDPro/main.html) [19,20]. The data from K2d are presented here.

### 2.6. Patient Sera

Sera were obtained from the blood of peanut allergic individuals, which were collected after informed consent at Tulane Health Science Center (New Orleans, LA, USA) in accordance with the rules and regulations of the institutional review board.

### 2.7. Western and Spot Blot Analysis with Patient Sera

Purified natural Ara h 1 (nAra h 1) [15] and rAra h 1 proteins (300 ngs) were either electrophoretically transferred to a nitrocellulose membrane following SDS-PAGE (western blot) using standard western blot procedure or spotted directly onto a PVDF membrane and allowed to dry. For IgE western blots and spot blots, membranes were blocked in 2% Blotto (2% dry milk dissolved into phosphate buffered saline (PBS) containing 0.5% TWEEN (PBST) for 30 min and incubated overnight with 1:10 dilution in PBST of patient sera from convincing history/documented peanut allergic individuals with positive skin prick test results. After the incubation patient sera, the membranes were washed 3 times with PBST and incubated with anti-human IgE conjugated to horseradish peroxidase (HRP)-labeled secondary antibody at 1:10,000, diluted in 2% blotto for 30 min. The membrane was then washed 3 times with PBST and 2 times with PBS and incubated with ECL-Plus Western substrate (Amersham Bioscience Corp., Piscataway, NJ, USA). The signal was then visualized using a CCD camera system (Fuji Photo Film Co., Ltd., Duluth, GA, USA).

## 3. Results and Discussion

### 3.1. Expression and Purification of Mature rAra h 1 

**Figure 2:**
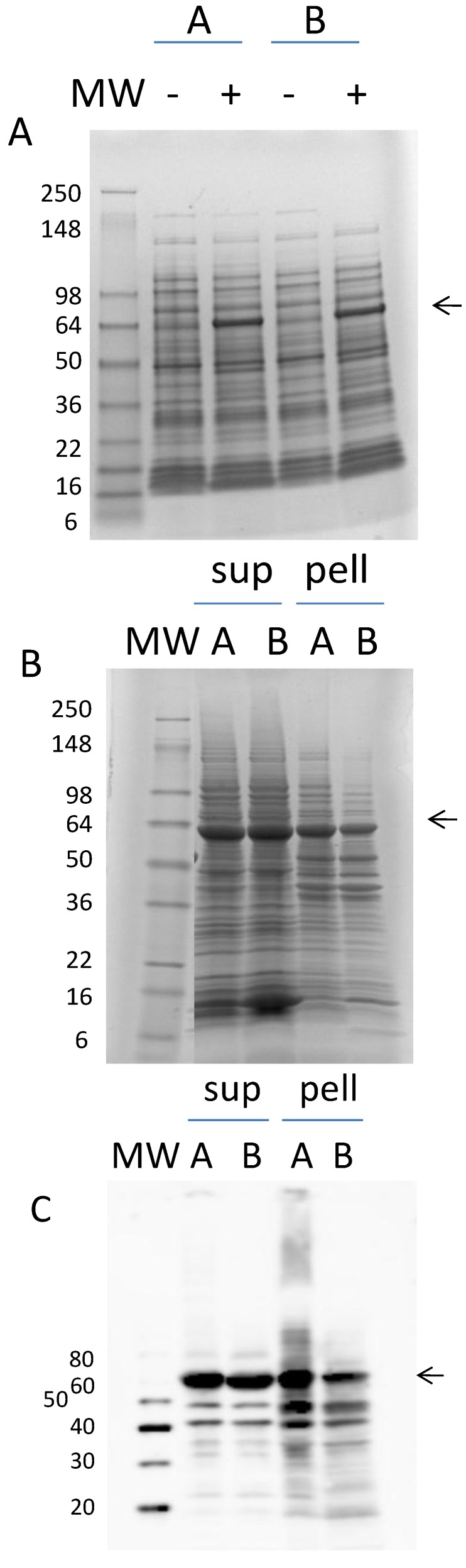
(**A**) SDS-PAGE of induced (+) and non-induced (−) cultures for clones A and B. The position of the induced mature rAra h 1 is indicated with an arrow. (**B**) SDS-PAGE of the soluble and insoluble fractions of induced cells (A and B clones) following lysis. “sup” is the soluble supernatant. “pell” is the insoluble cellular debris. (**C**) Western blot analysis with anti-Ara h 1 antibody of the same samples shown in B.

In Figure 1 is shown the full-length translation product for the Ara h 1 mRNA. The signal and leader peptides (red and blue) are removed to yield the mature, natural Ara h 1 protein (black and purple). The mature protein sequence was back translated using preferred codons for expression in *E. coli*, the gene synthesized and subsequently cloned into pET9a. Expression was tested on two clones (A and B) in strain BL21(DE3) pLysS. Following growth to mid-log phase and three-hour treatment with IPTG, virtually no induction was observed on SDS-PAGE gels of solublized whole cells (data not shown). However, reasonable induction was achieved with the same clones in BL21 (DE3) cells (Figure 2A). The arrow indicates the position of inducibly-expressed Ara h 1. Solubility of the protein was examined following lysis and centrifugation. The results are shown in Figure 2B. It appears that a little more than half of the expressed protein is in the soluble fraction (sup). In Figure 2C the identity of the expressed protein as Ara h 1 is confirmed by western blot analysis. There appears to be a few proteolytic cleavage products also in both the soluble and insoluble fractions.

**Figure 3:**
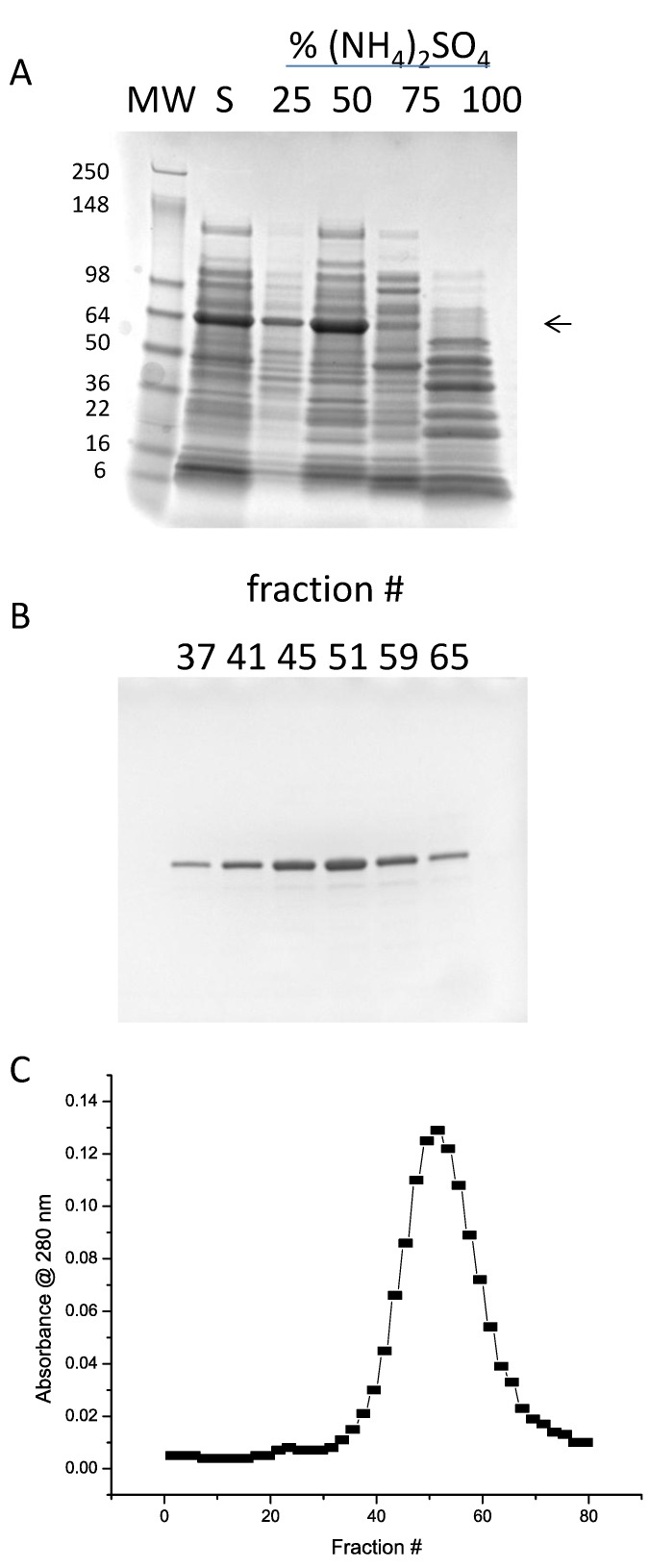
(**A**) SDS-PAGE of ammonium sulfate precipitation tests. “S” indicates the soluble fraction of lysed cells. (**B**) SDS-PAGE of column fractions from High-Q chromatography. (**C**) Elution profile of fractions from High-Q chromatography.

As a first step in purification, ammonium sulfate precipitation was tested. In Figure 3A is shown the soluble fraction and the results of bringing the soluble fraction to 25%, 50%, 75%, and 100% of saturation in a stepwise manner. It is clear that the vast majority of the Ara h 1 precipitates with 50% ammonium sulfate, with only a small amount at 25%. Therefore, a two-step ammonium sulfate procedure with an undercut of 25% (discarded) and cut of 50% was incorporated into the purification protocol. Various ion exchange resins were tested for binding and elution using the solublized 50% ammonium sulfate precipitate as the load. High-Q was found to have the best binding and elution characteristics. In Figure 3B,C are shown the SDS-PAGE and absorbance analysis of a gradient elution of rAra h 1. The shallow gradient was 100–250 mM NaCl. Over-loading SDS-PAGE gels with peak fraction samples showed small amounts of contaminating protein (not shown). The resultant preparation is estimated to be at least 95% Ara h 1. Peak fractions were pooled and stored at −20 °C.

### 3.2. Expression and Purification of Core rAra h 1 

**Figure 4:**
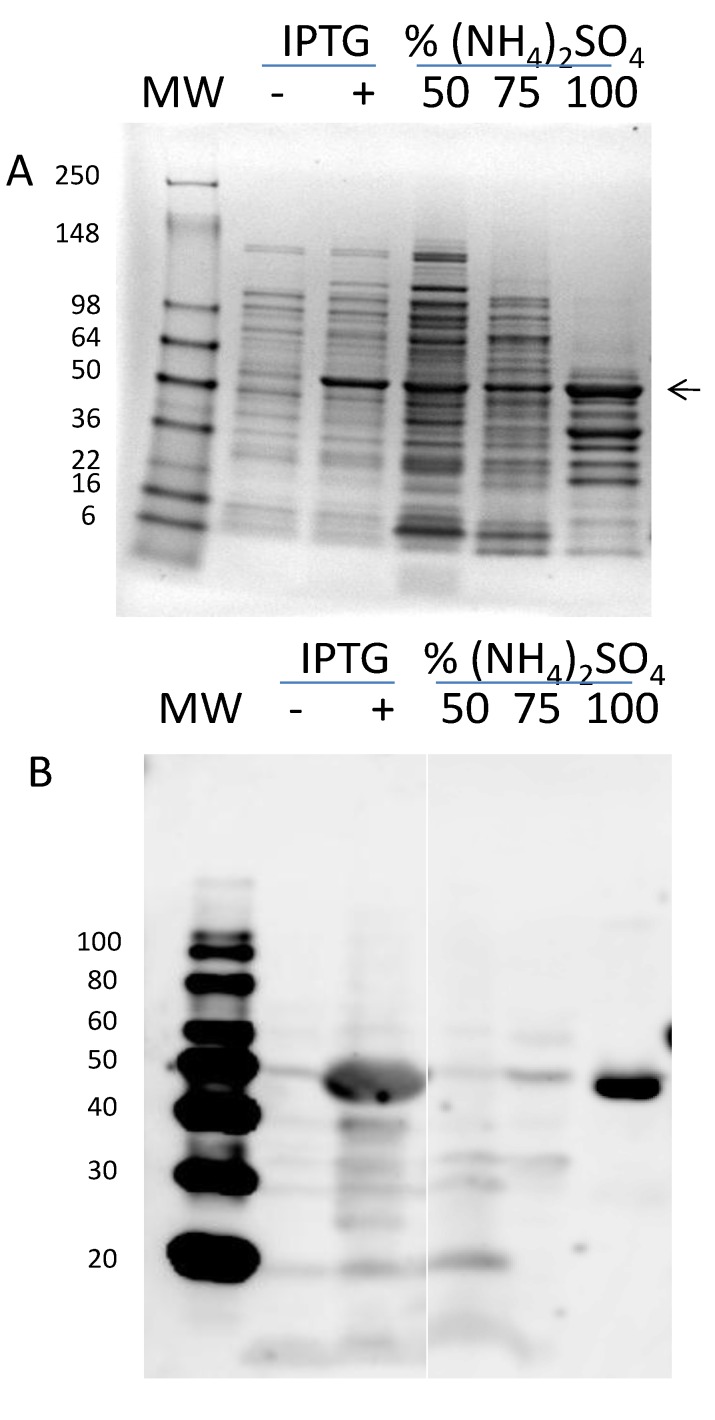
(**A**) SDS-PAGE of induced (+) and uninduced (−) cells and ammonium sulfate precipitations tests. (**B**) Western blot analysis of the same samples shown in (A).

The mature rAra h 1 purified by the procedure described above has been used for structural studies [13,14]. Neither group was able to obtain high-resolution crystals for structure determination. Therefore, we decided to make a second expression construct containing only the core region of Ara h 1 (black and purple in Figure 1). PCR was used to amplify codons 170–586 and the product was cloned into pET9a. In Figure 4A is shown the IPTG induction test samples and the stepwise ammonium sulfate precipitation tests. Expression was seen after three hours of induction. Western blot analysis (Figure 4B) confirmed the induced protein as rsAra h 1. Ammonium sulfate precipitation properties of this shorter protein were also tested. In Figure 4A, SDS-PAGE analysis shows a band of the appropriate size in the 50%, 75%, and 100% concentrations, with the majority in the 100%. Western blot analysis (Figure 4B) revealed that the co-migrating bands in the 50% and 75% samples were not Ara h 1 and nearly all of it is in the 100% ammonium sulfate sample. As a result, the purification protocol incorporated a 75% undercut and a 100% precipitation to collect the protein.

**Figure 5:**
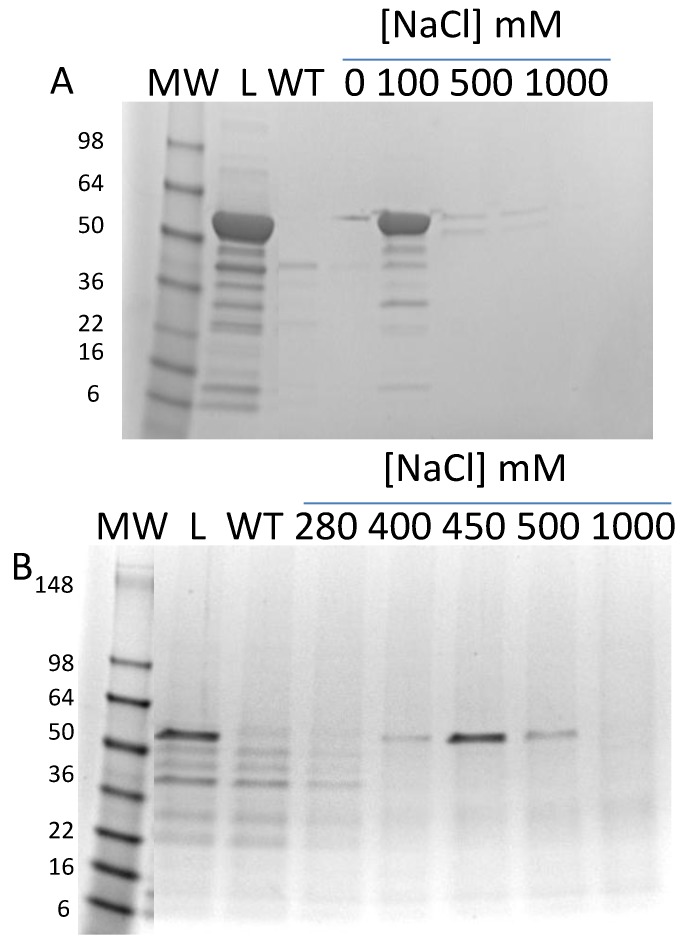
(**A**) SDS-PAGE of step gradient fractions from chromatography of rsAra h 1 on High-Q resin. “L” indicates the material loaded on the column. “WT” indicates the wash through material that didn’t bind the resin. (**B**) SDS-PAGE of step gradient fractions from chromatography of rsAra h 1 on High-S resin. “L” indicates the material loaded on the column. “WT” indicates the wash through material that didn’t bind the resin.

Various ion exchange resins were tested for binding and elution characteristics. In Figure 5A is shown the testing with High-Q resin. Core Ara h 1 would only bind this resin at a relatively high pH (8.4) and very low ionic strength (no salt). To take advantage of this, the purification protocol includes binding to High-Q in Q column buffer without salt and one-step elution with the same buffer with 100 mM NaCl. rsAra h 1 bound much more tightly to High-S than High-Q. However, to achieve binding the pH was reduced to 7.0. In Figure 5B is shown the step-wise elution of the protein. Little or no core protein eluted at 280 mM NaCl, but excellent elution occurred at 450 mM. Therefore, the next step in purification protocol became a load of the Q column eluate onto High-S after the pH was reduced and salt increased to 280 mM. A one-step elution with column buffer with 450 mM NaCl was applied.

**Figure 6:**
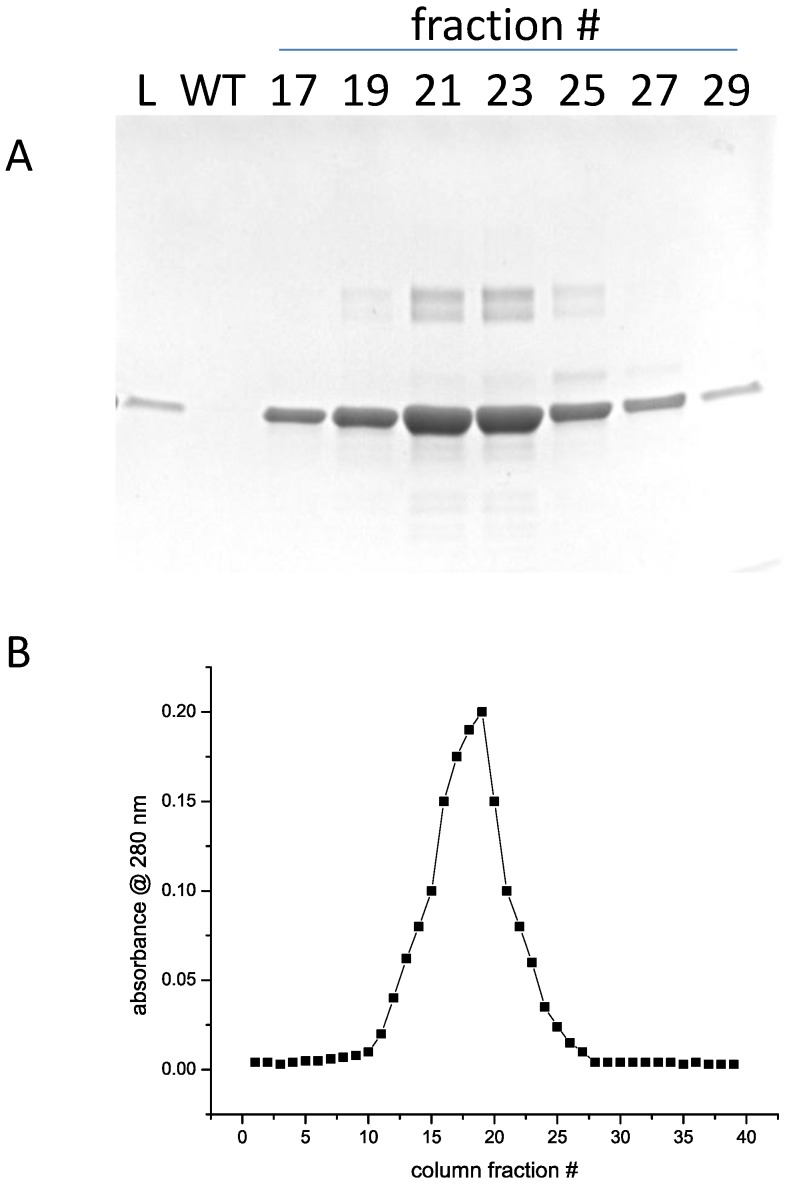
(**A**) SDS-PAGE of gradient fractions from chromatography of rsAra h 1 on High-S resin. “L” indicates the material loaded on the column. “WT” indicates the wash through material that didn’t bind the resin. (**B**) Elution profile of fractions from High-S chromatography.

The eluted protein was fairly dilute. To concentrate it, the High-S column was regenerated and equilibrated with S column buffer with 300 mM NaCl. The eluate from the previous column was diluted with buffer containing no salt to get a final NaCl concentration of 300 mM and loaded on the column. A steep gradient of 300 mM to 2 M NaCl was used to elute the core protein. In Figure 6A,B are shown the SDS-PAGE analysis and elution profile. Small amounts of higher molecular weight material can be seen in the highly-concentrated peak fractions. Peak fractions were pooled and stored at −20 °C.

The rAra h 1 and rsAra h 1 behaved very differently in the ammonium sulfate precipitations and on the ion exchange resins. This is likely due to a dramatic change in the charged amino acids. The core protein is missing 31 positively charge residue and 27 negatively charged residues. In general, larger proteins of complexes precipitate at lower concentrations of ammonium sulfate. The core Ara h 1 is 9 kDa smaller than the natural Ara h 1. We know that the core protein forms trimers because it is the protein used for structure determination [14]. However, it has also be reported that natural Ara h 1 can form even higher oligomeric structures [21].

### 3.3. Secondary Structure Analysis of Recombinant Mature and Core Ara h 1

**Figure 7:**
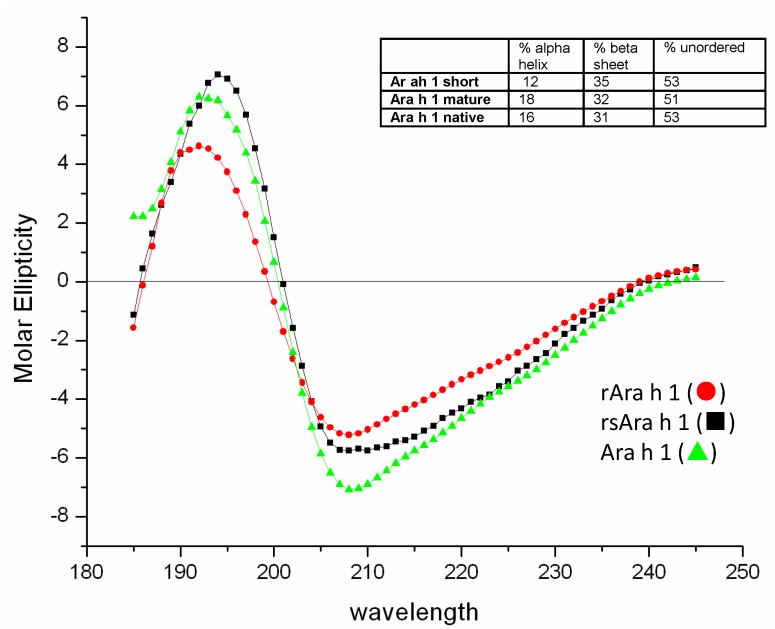
Circular dichroism analysis of rAra h 1 (●), rsAra h 1 (■) and natural of Ara h 1 (▲). The percentage of ordered and disordered secondary structure and shown in the inset.

Far-UV CD (185–260 nm) was used to compare the secondary structure content of mature rAra h 1, core rAra h 1 and natural Ara h 1 (Figure 7). The CD spectra show that the secondary structures of all three Ara h 1 forms are very similar exhibiting minimums at ~208 nm and ~222 nm and a crossover near 200, typical characteristics of proteins with either separate α + β region or combined α/β regions. However, the large 208:222 ratio is indicative of a structure with separate α and β regions, which corroborates the Ara h 1 trimeric models based on phaseolin and β-conglycinin [8] and crystal structures [13,14]. Further analysis of the CD spectra of recombinant and natural Ara h 1 using K2d shows that all three Ara h 1 forms contain similar secondary structure elements (Figure 7, inset) and the values obtained are valid according to normalized root mean square deviation values (NRMSD) that are used for internal software control and confirmed by other software, such as CDPro [22].

### 3.4. Western and Spot Blot Analysis

**Figure 8:**
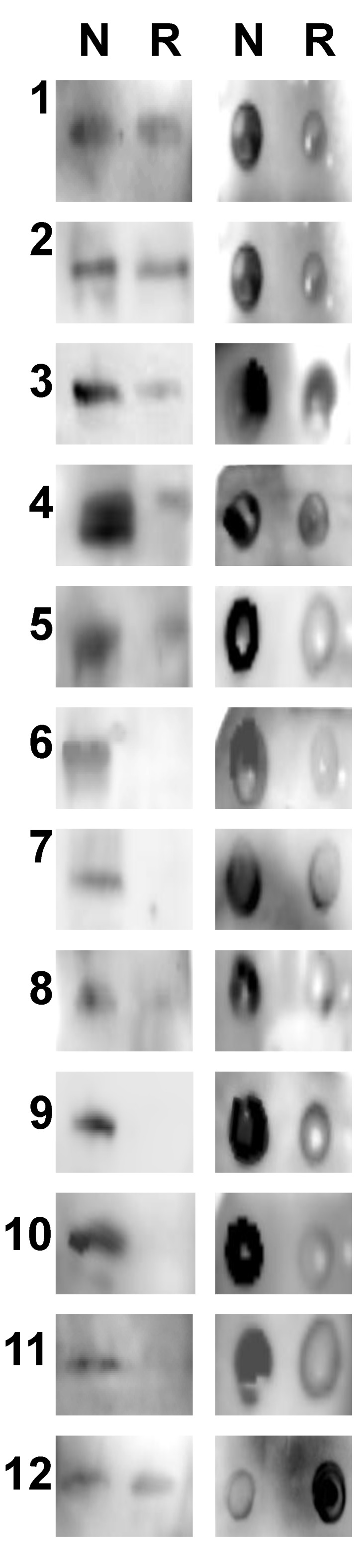
IgE western blot and spot blot comparison of natural and recombinant Ara h 1. Natural (N) and rAra h 1 (R) were subjected to western blot (left panels) or spot blot (right panels) with sera from 12 peanut allergic individuals (1 and 2).

Western blot (on left) and spot blot analysis (on right) were performed to compare IgE binding of 12 peanut allergic sera to natural Ara h 1 (N) *versus* rAra h 1 (R) in linear and folded form (Figure 8). Of the 12 patient sera tested IgE of all patients recognized the natural Ara h 1 in both folded and unfolded forms. In the western blots, five out of 12 (41%) of the sera (6, 7, 9, 10, and 11) did not recognize the linear rAra h 1 at all or at very low levels. The IgE binding was significantly reduced for five (41%) of the sera (3, 4, 5, 8, and 12) and only two (16%) did not show a significant difference in binding (1 and 2). In the spot blot analysis all patient sera recognized both N and R Ara h 1 in folded form, but the IgE binding was significantly reduced for all patient sera with the exception of serum 12, which had increased binding to R compared to N.

## 4. Conclusions

While production of recombinant Ara h 1 has been briefly described in other publications [12,13,14], the methods were laborious and inefficient. Here, we’ve optimized the DNA clone, the expression and purification for a highly efficient large-scale production of these proteins and mutants thereof without the need for any costly instrument requirements. Purification procedures described here will allow production of sufficient quantities of pure proteins with which to undertake further structural and immunological studies to understand the allergic response better. Here the folded and unfolded forms of the recombinant Ara h 1 showed lower IgE binding by the majority of patient sera tested, which indicates that while it might not be as useful as the natural Ara h 1 for diagnostics, it may provide a safer immunotherapeutic tool. The loss of reactivity with rAra h 1 *versus* natural Ara h 1 in western blot inplicates reactivity with post-translational modifications, namely advanced glycation end products [23]. Importantly, we intend to begin modifying amino acids identified from studies that have mapped and mutated the linear IgE epitopes. Those modified proteins will be tested for IgE binding. Hypoallergenic Ara h 1 could be a very important tool in our overall goal of reducing allergy to peanut.
